# Notes and News

**Published:** 1897-07-03

**Authors:** 


### Notes and News.

(Collected and Communicated.)

Lord Lister has not accepted an invitation to sit upon the Council of the Royal College of Surgeons.

The children attending the Board schools at Newcastle have subscribed to maintain two cots at the Fleming Memorial Hospital for one year.

The Middlesex and St. George's Hospitals have each received £10,000 through the will of the late Mr. David Brandon.

The new hospital of the Metropolitan Asylums Board, the Park Hospital at Hither Green, will be opened by the Princess of Wales on July 12th.

Alderman Reeves, of Brighton, has presented the Sussex County Hospital with two houses at Ditchling for the use of convalescent patients.

The latest convalescent home in connection with the Birmingham Hospital Saturday Fund, Marle Lodge, has been opened for women at Llandudno.

Messrs. Hope Brothers devoted the profits arising from the letting of seats to view the Jubilee procession to the Prince of Wales's Hospital Fund, thus contributing no less a sum than £613.

The bronze statue of the late Sir Richard Owen, subscribed for by his friends and admirers and executed by Mr. Thomas Brock, R.A., is now placed in the Natural History Museum at South Kensington. It stands upon a Numidian marble pedestal, provided by the trustees of the British Museum.

The Duke of Westminster, as President, has fixed Saturday, July 10th, for "Queen's Day," in the National Hospital for the Paralysed and Epileptic (Albany Memorial). The Duchess of Albany and the Duchess of York have promised to be present, if possible. The programme will include an inspection of the wards, a musical conversazione in the afternoon, arranged by Madlle. Janotha; and in the evening a Dream of Flowers, by Miss Allanson-Winn.

H.R.H. the Princess Christian opened the New General Hospital, Birmingham, on Wednesday. She was accompanied by Prince Christian.

The ladies of Leeds have done a good work for the medical charities of the town, resulting in a collection of £756 during the past year. A doll and toy show served to considerably augment the Ladies' Hospital Fund on the present occasion.

The reports as to the successful methods of inoculation for rinderpest are certainly conflicting. In some directions Koch's serum is said to be successful, and in others that used by Dr. Edington is regarded as far more effective.

The annual general meeting of the members of the British Medical Association will be held in Exeter Hall on Tuesday, July 27th. The scientific business of the association will be transacted at Montreal as already announced.

Mr. Maurice Beddington, of Carshalton, has presented a site of four and a half acres in extent for the erection of a convalescent home for women as a Jubilee commemoration for Surrey. The site is close to High Street, Ramsgate, on high ground, and near two railway stations.

A new hospital for ear, throat, and eye cases was recently opened at Washington, in the United States. The first floor is arranged for the out-patients and dispensary. The second floor is devoted to private patients and offices; the third floor contains free wards; and the fourth is devoted to coloured patients. The operating-room is on the third floor.

A stained-glass window has been placed in a church at Fernandina, in America, in memory of two medical heroes. Yellow fever raged in Fernandina in 1877, numbers dying for want of medical aid. Drs. Welford and Herndon, doctors in Virginia, came to the town and devoted themselves to the alleviation of the condition of the stricken inhabitants, until they themselves became victims of the fever.

We congratulate the Sisters of St. Margaret's, East Grinstead, upon the issue of the first annual report of the National Free Home for the Dying at Clapham, which is under their charge. We thoroughly endorse the remark of a visitor to the institution that it is more like a home than a hospital, and this, no doubt, would be still more the case were funds forthcoming to enable it to move into more suitable quarters.

Very bad accounts come from Cuba respecting the health of the troops. Twenty thousand sick are reported to be in the hospitals. The heat is intense, and yellow fever and dysentery especially are raging. Illness has attacked the medical staff, and the number of medical men is all too few to cope with the work. There are numbers of vacancies on the staff, and there is no appearance of any improvement in the general conditions being likely to take place at present.

